# Traffic Vibration Signal Analysis of DAS Fiber Optic Cables with Different Coupling Based on an Improved Wavelet Thresholding Method

**DOI:** 10.3390/s23125727

**Published:** 2023-06-19

**Authors:** Yuhang An, Jihui Ma, Tuanwei Xu, Yunpeng Cai, Huiyong Liu, Yuting Sun, Wenfa Yan

**Affiliations:** 1 Key Laboratory of Transport Industry of Big Data Application Technologies for Comprehensive Transport, Ministry of Transport, Beijing Jiaotong University, Beijing 100044, China; 21114020@bjtu.edu.cn (Y.A.); jhma@bjtu.edu.cn (J.M.); sunyuting@bjtu.edu.cn (Y.S.); yanwenfa@bjtu.edu.cn (W.Y.); 2 State Key Laboratory of Transducer Technology, Institute of Semiconductors, Chinese Academy of Sciences, Beijing 100083, China; 3 College of Materials Science and Opto-Electronic Technology, University of Chinese Academy of Sciences, Beijing 100049, China; 4 School of Information Management, Beijing Information Science and Technology University, Beijing 100192, China; liuhy@bistu.edu.cn

**Keywords:** distributed acoustic sensing (DAS), vibration signal, deployment methods, cable coupling, wavelet threshold algorithm

## Abstract

Distributed Acoustic Sensing (DAS) is a novel technology that uses fiber optics to sense and monitor vibrations. It has demonstrated immense potential for various applications, including seismology research, traffic vibration detection, structural health inspection, and lifeline engineering. DAS technology transforms long sections of fiber optic cables into a high-density array of vibration sensors, providing exceptional spatial and temporal resolution for real-time monitoring of vibrations. Obtaining high-quality vibration data using DAS requires a robust coupling between the fiber optic cable and the ground layer. The study utilized the DAS system to detect vibration signals generated by vehicles operating on the campus road of Beijing Jiaotong University. Three distinct deployment methods were employed: the uncoupled fiber on the road, the underground communication fiber optic cable ducts, and the cement-bonded fixed fiber optic cable on the road shoulder, and compared for their outcomes. Vehicle vibration signals under the three deployment methods were analyzed using an improved wavelet threshold algorithm, which was verified to be effective. The results indicate that for practical applications, the most effective deployment method is the cement-bonded fixed fiber optic cable on the road shoulder, followed by the uncoupled fiber on the road, and the underground communication fiber optic cable ducts are the least effective. This has important implications for the future development of DAS as a tool for various fields.

## 1. Introduction

The advancement of science and technology has led to a significant focus on intelligent transportation in transportation research. ITSs combine various technologies, including electronics, information technology, sensors, and system engineering, to monitor and manage traffic within the road infrastructure [1]. Real-time intelligent monitoring of traffic flow and providing a basis for command decisions in traffic management are essential [2]. For intelligent traffic management, accurate and efficient detection of traffic flow is crucial. The five primary categories that classify traffic detection technologies are radar [3,4], infrared [5,6], magnetic [7,8], video [9,10], and wireless sensor network detection technology [11,12]. However, conventional detection methods possess particular limitations. Distributed Acoustic Sensing technology was used in this study for traffic detection. The development of fiber optic sensing technology dates back to 1977, and it has progressed rapidly since then, paralleling the development of fiber optic communication technology. The degree of informatization of a country is often measured by its progress in this technology. DAS is an emerging fiber optic sensing technology that uses the entire fiber both as the sensing medium and transmission signal medium [13]. Through the measurement of specific scattered light signals, DAS detects changes in its surrounding environment or the fiber itself. It is a fully distributed measurement technology that incorporates both sensing and transmission, making it capable of long-range measurement and monitoring [14]. With a single measurement, it can acquire spatial parameters of the entire fiber distribution area and measure information up to tens or hundreds of kilometers away. Fiber optic sensing technology has been widely applied across various fields, including military, national defense, aerospace, industrial and mining enterprises, energy and environmental protection, industrial control, medicine and health, measurement and testing, construction, household appliances, and more. Its versatility has created a broad application market. Distributed acoustic sensing technology for traffic detection offers various advantages over traditional traffic detection technology. These advantages include affordability, compactness, corrosion resistance, tolerance to high temperatures and voltages, immunity to electromagnetic interference, sensitivity, and covertness [15].

Several studies have shown that distributed acoustic sensing technology is suitable for real-time monitoring of traffic behavior. Wang and Zeng [16] utilized DAS technology to record signals from urban traffic, seismic background noise, and artificial seismic sources successfully. Moreover, they accomplished near-surface structural imaging by implementing the technology in urban communication fiber optic cables. Revised sentence: Song and Zeng [17] employed DAS technology, in combination with a highly reproducible air gun source, to construct a system for monitoring the high-resolution urban subsurface. The system provides frequent snapshots of near-surface in urban areas. Revised sentence: In addition, Zeng et al. [18] demonstrated the feasibility of using DAS and 7.6 km of dark fiber in Tangshan, China, to detect seismic activity after the 12 July 2020, 5.1 magnitude earthquake. The experimental results indicated that post-earthquake monitoring using DAS and dark fiber reduces costs and time and helps mitigate disasters by capturing more aftershocks. Martin N. Lindsey, Shan et al. [19] conducted a study on the sources of a waveform in Distributed Acoustic Sensing equipment deployed on a highway situated north of Fairbanks, Alaska. Their research revealed that cars driving on adjacent roads were the primary source of noise on the highway. Cai et al. [20] proposed a DAS-based aircraft acoustic signal detection system and analyzed the acoustic characteristics of aircraft signals for continuous monitoring of aircraft off-airports. Experimental tests were conducted using an underground communication fiber that was 8.1 km long. Results showed that the seismic vibration signals, excited by the aircraft acoustics, had frequencies below 5 hertz and were primarily low-frequency signals. Liu et al. [21,22] proposed a vehicle detection and classification system that deployed DAS technology for detecting, classifying, and estimating vehicle speed. The cement mortar fixed the fiber optic at the mine in Nanshan and the campus of Beijing Jiaotong University. The validation data, collected using distributed fiber optic acoustic sensors, showed that the vehicle detection accuracy was more than 80%. The speed estimation error was below 5%, and the vehicle classification accuracy exceeded 70%. Cai et al. [23] used Distributed Acoustic Sensing technology to detect campus security events by conducting field experiments. They monitored several aspects including emergency detection, vehicle localization, speeding alarms, and congestion alarms. The results showed that the detection accuracy of the system was high, thus indicating the practical significance of this approach. These experiments proved the potential of DAS for real-time monitoring of traffic behavior.

In the experimental setup of the aforementioned studies, optical fibers were utilized in various deployments. The coupling impact varies depending on the deployment, and the acquired data quality also varies. Optical fiber coupling can significantly improve the efficiency and accuracy of detection results. This is especially important in the field of transportation, where it can provide comprehensive solutions for urban traffic flow detection, structural health detection, traffic safety monitoring, early warning systems, and perimeter security detection, among others, with real-time accuracy. Enhancing our daily lives with superior convenience. Currently, acquiring high-quality vibration data is the main focus of research. The fiber structure and deployment method are two important factors that affect the performance of the DAS system. To acquire high-quality vibration data, it is crucial to improve the coupling quality between the fiber optic cable and the ground layer of the DAS system. Therefore, investigating methods for improving the coupling quality is essential [24,25,26]. While research on different burial depths of optical fibers is available, there are limited studies on the deployment of traffic signals using different deployment methods of fiber optics. In November 2018, the Fiber Optic Sensing Association published Installation Considerations for Highways for Fiber Optics [27], which compared the performance of various fiber deployment methods. However, it only provided a rough assessment without providing detailed data analysis of each method’s performance. Moreover, it did not consider comparing the underground communication fiber optic cable ducts. The purpose of this study is to compare vibration signal data obtained from three deployment methods with the use of DAS technology to overcome existing monitoring method limitations. The study found that the cement-bonded fixed fiber optic cable on the road shoulder provided the best vibration signal quality, which sheds light on DAS technology’s deployment in transportation.

The present paper is structured as follows: Section 2 provides an introduction to distributed fiber optic sensors. Section 3 presents the detecting algorithm, and Section 4 details the experimental setup. Section 5 analyzes the experimental results. Finally, Section 6 concludes the paper and discusses limitations and future issues that require further attention.

## 2. Distributed Fiber Optic Sensors

**Figure 1:**
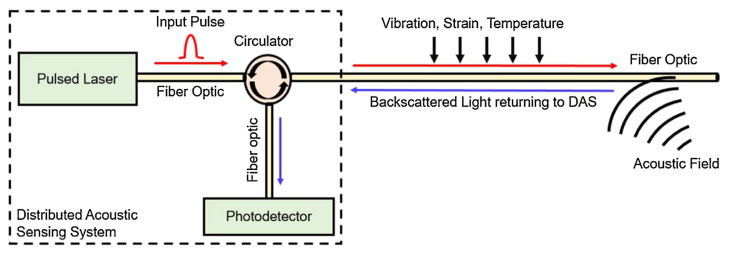
DAS schematic diagram.

Distributed Optical Fiber Sensors (DOFS) rely on changes in the physical properties of light waves transmitted through optical fibers to detect external stimuli, including temperature, pressure, strain, and vibration. The schematic diagram of the Distributed Acoustic Sensing system is depicted in Figure 1. DOFS can be classified into grating-based, backscattered light-based [28], and interference-based sensing methods based on sensing principles. Currently, one of the more mature techniques is the Optical Time-Domain Reflectometer (OTDR) based on Rayleigh scattered light [29]: it uses the entire optical fiber as the sensing medium and for transmitting signals. The Optical Time-Domain Reflectometer uses the properties of backscattered light, including light intensity, polarization state, phase change, and other related parameters, to change the sensing parameters. This, in turn, allows for measuring external physical quantities such as vibration signals [30]. In 1993, H. F. Taylor et al. [31], introduced the Rayleigh scattering-based phase-sensitive optical time-domain reflectometer (Φ-OTDR) technique and applied it to detecting vibration signals. The Φ-OTDR employs a narrow-band laser source that enables the analysis of the optical power of the backscattered light generated by interference along the time axis distribution profile [32]. Subjected to external perturbations, an optical fiber’s position and refractive index are deformed, causing changes to the signal’s phase and amplitude. Analyzing changes in the Rayleigh scattering curve before and after a disturbance allows for detecting dynamic events, such as vibration signals present in structures. Distributed vibroacoustic sensing using Φ-OTDR technology offers benefits such as long sensing distance, high spatial resolution, high sensitivity, wide dynamic range, ability to work in adverse conditions, and respond quickly [33]. In this study, utilizing a DAS system based on Φ-OTDR technology, we collect vibration signals from traffic and analyze the vibration signal quality of three different DAS optical cable couplings.

**Figure 2:**
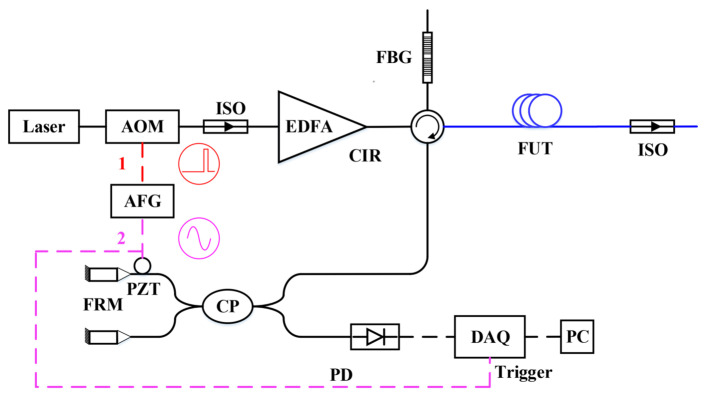
Scheme of DAS system based on PGC algorithm.

Figure 2 shows the system scheme of the distributed acoustic sensing system used in this paper, which is based on the phase-generated carrier algorithm and Φ-OTDR technology. The system comprises a laser source (Laser), an acousto-optic modulator (AOM), an Erbium-doped fiber amplifier (EDFA), a circulator (CIR), a fiber Bragg grating (FBG), a Michelson interferometer, a photodetector, and a data acquisition and signal processing module, as shown in Figure 2. The narrow linewidth continuous-wave laser output is modulated by the AOM to generate pulse light (pulse width: 50 ns, repetition frequency: 10 kHz). The pulse light enters the EDFA via an isolator to complete the optical amplification process. The amplified pulse light is transmitted through the circulator into the FBG to eliminate the forward ASE and spontaneous emission noise from the EDFA. Subsequently, the light is transmitted into the sensing fiber through the three ports of the circulator, and the backward Rayleigh scattering from the sensing fiber is transmitted through the fourth port of the circulator into the Michelson interferometer. The Michelson interferometer comprises a coupler and two Faraday rotation mirrors (FRMs), which rotate the polarization state of the incident light by 45 degrees each. The FRMs in the Michelson interferometer produce a 90-degree polarization change, compensating for the polarization changes in both directions and addressing the polarization fading problem. The coherent Rayleigh scattering light output from the interferometer is detected by the photodetector and then converted into an electrical signal via a data acquisition card. The signal processing unit performs the phase demodulation along the fiber link through the phase-generated carrier (PGC) algorithm. Figure 2 shows a pulse signal as 1 and a sinusoidal signal as 2, allowing us to distinguish between the two signals.

The PGC demodulation algorithm [34] was utilized to recover the phase information of the sensing information propagating along the optical fiber. The interference signal was split and separately multiplied by both a fundamental carrier and a second-harmonic carrier of the PGC signal. The resulting signal was filtered using two identical low-pass filters (LPF) to remove undesirable high-frequency spectrum. The cut-off frequency of the two low-pass filters is crucial and should be less than half of the PGC signal frequency, generating the in-phase and quadrature components. Then, the arctangent operation was used to calculate the phase. Next, a filter was designed to filter out the low-frequency shift of the phase, improving the signal-to-noise ratio of the sensing information.

## 3. Detection Algorithm

**Figure 3:**
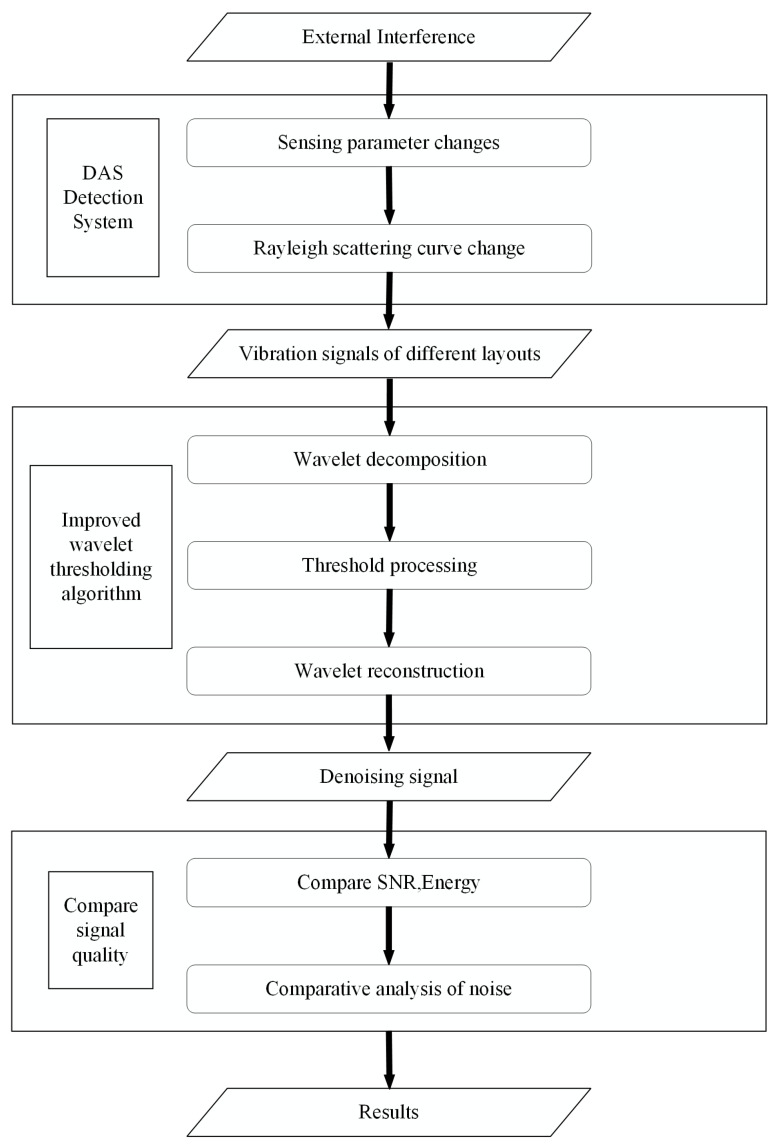
Summary of the complete workflow.

This study employs an improved wavelet threshold algorithm for vehicle detection at traffic signals. Distributed acoustic sensing technology is utilized to acquire vehicle vibration data, which serves as the foundation for further analysis. The wavelet thresholding algorithm is then applied to denoise the data obtained by DAS. A comparative analysis of signal data acquired through three distinct optical fiber deployment methods is conducted to determine the optimal vehicle detection method. The flow chart of the detection process is depicted in Figure 3.

### 3.1. Improved Wavelet Thresholding Algorithm

The selection of the threshold function is critical in constructing the continuity and accuracy of the signal and has a significant impact on the effectiveness of wavelet denoising. Currently, there are two primary methods for selecting the threshold, namely: the hard threshold and the soft threshold. The hard threshold method compares the absolute value of the signal with the threshold, sets the signal that is less than or equal to the threshold to zero and retains the signal that is greater than the threshold. However, the discontinuity of the hard threshold method results in obvious noise in the denoised signal. On the other hand, the hard threshold is capable of retaining the local information of the image edges and details while producing visual distortions such as the ringing effect. Unlike the hard threshold, the soft threshold method sets the signals whose absolute value is smaller or equal to the threshold to zero, and for the signals greater than the threshold, it sets them as their own difference with the threshold, thereby allowing the signals to shrink towards zero. Although the soft threshold has better continuity than the hard threshold, its derivative is discontinuous. Consequently, it is estimated that there is a constant deviation between the noisy and denoised wavelet coefficients. Additionally, the fixed value compression of coefficients greater than the threshold and the decrease in noise with increasing wavelet coefficients are inconsistent with the facts. As a result, while the soft threshold technique produces relatively smoother denoised signals, it may also lead to blurry edges and distortion. The mathematical expressions for these two threshold functions are presented in the table below.

The equation of the hard thresholding method is as follows.

(1) $$\omega =\{\begin{array}{c} \;\;0,\;\;\;\;\;\;\;\;\left|\omega_{1}\right|<\lambda \;\\ \omega_{1},\;\;\;\;\;\;\;\;\left|\omega_{1}\right|\geq \;\lambda \; \end{array}$$

where ω is the estimated wavelet coefficient, $\omega_{1}$ is the wavelet coefficient and λ is the given threshold value.

The soft thresholding method is formulated as follows.

(2) $$\omega =\{\begin{array}{c} \;\;\;\;\;\;\;\;\;\;\;\;\;\;\;\;\;\;\;\;\;\;\;\;\;\;\;\;0,\;\;\;\;\;\;\;\;\left|\omega_{1}\right|<\lambda \\ sgn\left(\omega_{1}\right)\left|\omega_{1}-\lambda \right|,\;\;\;\;\;\;\;\;\left|\omega_{1}\right|\geq \lambda \end{array}$$

where sgn() is a symbolic function.

While traditional hard and soft thresholding methods have demonstrated their effectiveness in real-world applications, these methods come with their own potential drawbacks. Specifically, ω obtained using the hard thresholding method is discontinuous at ±λ. Consequently, reconstructing the signal utilizing this discontinuous ω can lead to the Pseudo-Gibbs phenomenon, causing oscillations in the final signal at locations of discontinuity or rapid change. Similarly, though ω obtained via soft thresholding has good continuity and produces a relatively smooth denoised signal, ω and $\omega_{1}$ always exhibit a constant bias, which can cause the signal to be excessively smooth at certain peak points, thus losing some original signal features. Overall, using either hard or soft thresholding methods for denoising is suboptimal when it comes to noise reduction.

This study uses an improved wavelet thresholding function proposed by group member Liu [21,22] to address the limitations of conventional thresholding denoising. The function is defined by the following equation [21]:

(3) $$\omega =\{\begin{array}{c} \;\;\;\;\;\;\;\;\;\;\;\;\;\;\;\;\;\;\;\;\;\;\;\;\;\;\;\;\;\;\;\;\;\;\;\;\;\;\;\;\;\;\;\;\;\;\;\;\;\;\;\;\;\;\;\;\;\;\;\;\;\;\;\;\;\;\;\;\;\;\;\;\;0,\;\;\;\;\;\;\left|\omega_{1}\right|<\lambda \\ \frac{1}{e^{\frac{\left|\omega_{1}-\lambda \right|}{a}}}sgn\left(\omega_{1}\right)\left|\omega_{1}-\lambda \right|+\left(1-\frac{1}{e^{\frac{\left|\omega_{1}-\lambda \right|}{a}}}\right)\omega_{1},\;\;\;\;\;\;\left|\omega_{1}\right|\geq \lambda \end{array},$$

where a is an adjustment factor. The value of a adjusts the overall convergence of the function to the speed of the original wavelet coefficients. When a tends to 0, the improved wavelet threshold function becomes a hard threshold function; when a tends to infinity, the improved wavelet threshold function becomes a soft threshold function.

The improved wavelet threshold function overcomes the limitations of hard thresholding, which is limited by its discontinuity at the threshold. Additionally, the improved smoothness of the reconstructed signal mitigates the drawbacks of using a soft threshold. The denoising algorithm that employs the improved wavelet threshold function consists of three steps: specifically, decomposing the wavelet signal, thresholding its coefficients, and reconstructing the signal after thresholding. The effectiveness of the algorithm is demonstrated through numerous simulation experiments.

### 3.2. Simulation Experiments of the Improved Thresholding Denoising Algorithm

**Figure 4:**
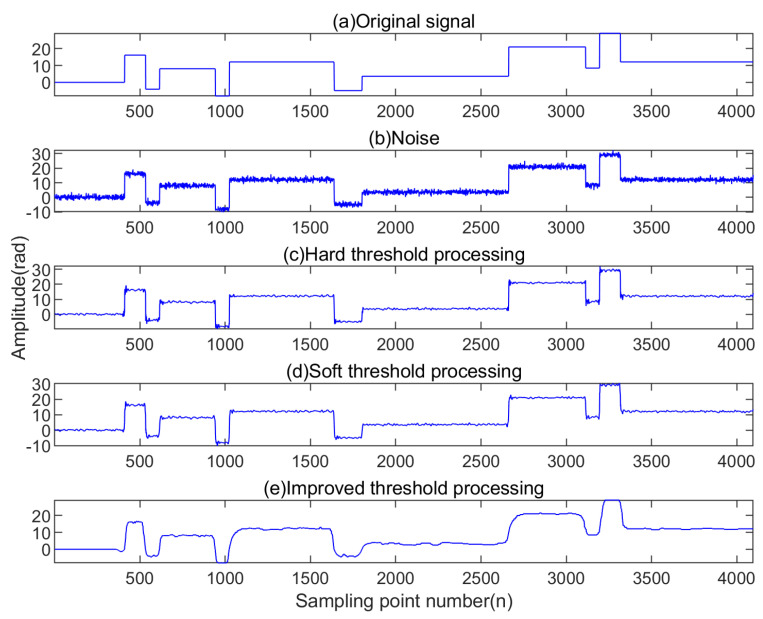
Signal denoising results of several methods.

The original signal displayed in Figure 4 was simulated. We added white noise to the original signal using MATLAB simulation software. Next, we subjected the resulting signal to three thresholding methods: hard, soft, and improved threshold. These methods were used to evaluate the effectiveness of the improved threshold function in the wavelet denoising algorithm. Three thresholding algorithms were implemented in MATLAB, including hard thresholding, soft thresholding, and improved wavelet thresholding. The computation times for the three methods for the same data set and on the same machine were recorded, with hard thresholding taking 0.17732 s, soft thresholding taking 0.17758 s, and improved wavelet thresholding taking 0.17798 s.

Figure 4 shows the results of the simulated denoising process using hard, soft, and improved thresholding methods. Specifically, the figures demonstrate that hard and soft thresholding led to different degrees of waveform distortion, while the improved thresholding algorithm achieved superior denoising outcomes.

Furthermore, to quantify the denoising outcomes, we assess the denoising algorithm using two metrics: signal-to-noise ratio (SNR) and root mean square error (RMSE). The formulas can be expressed as:

(4) $$SNR_{1}=10lg\frac{\sum_{n}s{\left(n\right)}^{2}}{\sum_{n}{\left(s\left(n\right)-\hat{s}\left(n\right)\right)}^{2}}$$

(5) $$RMSE=\sqrt{\frac{1}{n}\sum_{n}{\left(s\left(n\right)-\hat{s}\left(n\right)\right)}^{2}}$$

where s(n) is the original signal, $\hat{s}\left(n\right)$ is the estimated signal after wavelet threshold denoising, and *n* is the sampling point.

Table 1 summarizes the experimental results of denoising signals using different thresholding methods. The results in Table 1 indicate improvements in signal-to-noise ratio and reductions in root-mean-square error were observed with the improved thresholding method, compared to traditional thresholding methods. Therefore, the improved threshold denoising method achieved superior denoising outcomes.

Figure 4 and Table 1 show that the improved wavelet threshold denoising signal successfully reconstructed the signal peak with smooth features. The combined use of the improved threshold denoising and the traditional wavelet threshold denoising exhibited several advantages, resulting in a superior filtering effect when compared to using traditional wavelet threshold denoising alone.

## 4. Experimental Setup

**Figure 5:**
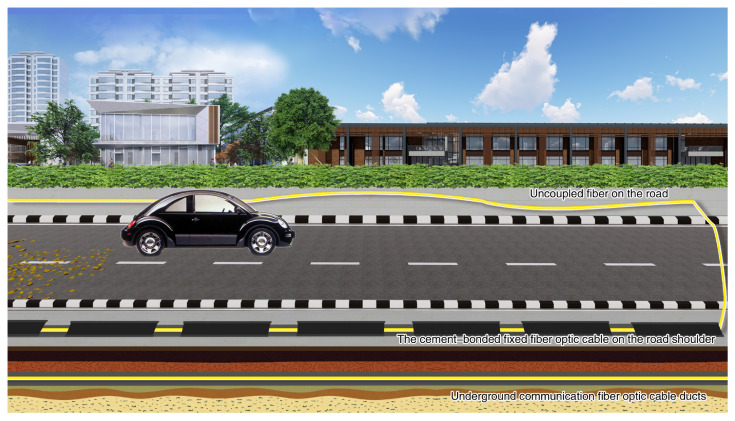
Schematic diagram of the fiber deployment method.

**Figure 6:**
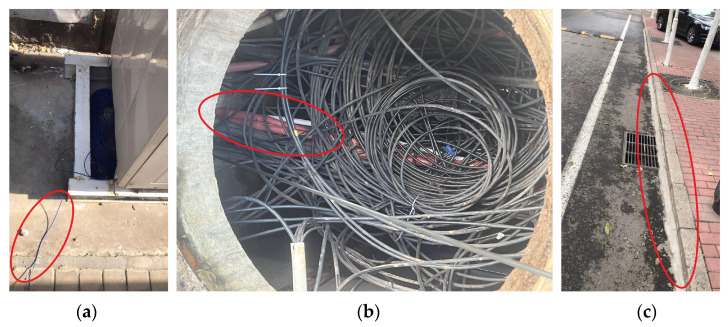
The photo with the cable layout: (**a**) the uncoupled fiber on the road; (**b**) the underground communication fiber optic cable ducts; (**c**) the cement-bonded fixed fiber optic cable on the road shoulder.

Experiments were conducted at Beijing Jiaotong University to validate the algorithm’s efficacy and compare the quality of detected signals in the distributed acoustic sensing system deployed under different methods. The DAS system’s laser pulse has a duration of 50 nanoseconds, and a pulse repetition rate of 10 kHz The data acquisition sensors were optical fibers. In the experiment, field-deployed common armored sensing optical fiber and communication optical cable laid in the underground cable trench on the campus of Beijing Jiaotong University were used. The DAS equipment used in the experiment was provided by the Optoelectronic System Laboratory of the Chinese Academy of Sciences Semiconductor Research Institute. The model of the DAS equipment used in this study is the SemiDAS-S001 model, manufactured by the Chinese Academy of Sciences Semiconductor Research Institute. The fibers were deployed using various methods in different locations such as the Information Center, Dormitory 19, Dormitory 8, and Jixiu Road. Three distinct methods were used to lay the optical fibers on the Beijing Jiaotong University campus. These methods included uncoupled fiber on the road, underground communication fiber optic cable ducts, and cement-bonded fixed fiber optic cable on the road shoulder. During the experiment, the recorded channels were numbered from 90 to 480, with every 30 s constituting a group of vehicle detection data and each channel covering 1.6 m. The illustration in Figure 5 demonstrates three different fiber deployments. The fiber exposed in the air is the uncoupled fiber on the road with a distance of 3.75 m from the road’s center, and the fiber covered by the cement block is the underground communication fiber optic cable ducts with a distance of 4.01 m from the road’s center, and the fiber in the soil layer is the cement-bonded fixed fiber optic cable on the road shoulder with a distance of 3.52 m from the road’s center. A total of 480 fiber optic channels were laid out, out of which 390 channels were recorded. Among the recorded channels, the ones for the cement-bonded fixed fiber optic cable on the road shoulder ranged from 95 to 170. On the other hand, the uncoupled fiber on the road had channels numbered between 171 and 250, while the underground communication fiber optic cable ducts had channels numbered between 380 and 475. Figure 6 provides a visual representation of the cable layout wherein the left figure illustrates the cement-bonded fixed fiber optic cable on the road shoulder, the middle figure depicts the uncoupled fiber on the road, and the right figure depicts the underground communication fiber optic cable ducts.

**Figure 7:**
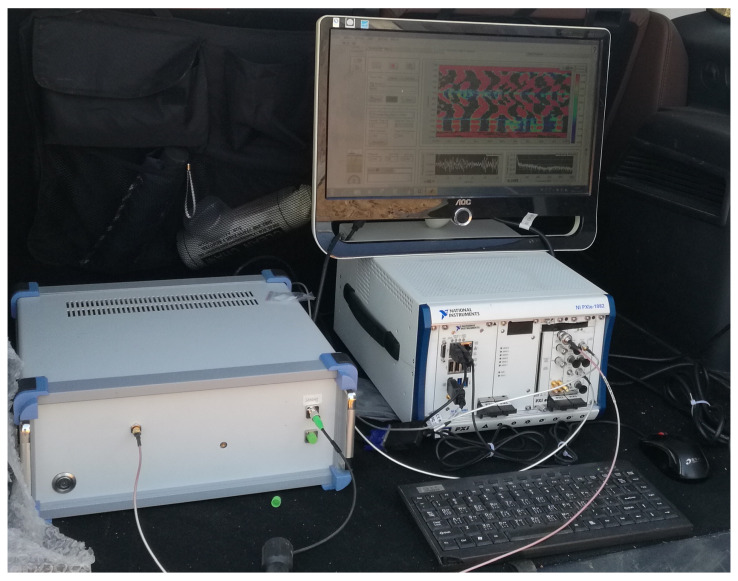
DAS system software and hardware platform.

Figure 7 illustrates the DAS system’s hardware and software platform. The hardware system is located below, while the software system is situated above. The DAS equipment used in the experiment was provided by the Optoelectronic System Laboratory of the Chinese Academy of Sciences Semiconductor Research Institute. The model of the DAS equipment used in this study is the SemiDAS-S001 model, manufactured by the Chinese Academy of Sciences Semiconductor Research Institute. During the optical fiber installation, the fiber was connected to the DAS host at one end, while the other was bent. The DAS system employs a time interval of 0.2 ms to collect data at each acquisition point, resulting in 5000 data samples per second. The unit utilized by the DAS system is the phase difference. The DAS system’s laser pulse has a duration of 50 nanoseconds and a pulse repetition rate of 10 kHz. The study recorded the vibration signals of vehicles passing by. The detection area comprised 390 sampling points, and the interval between sampling points was 1.6 m, with a sampling frequency of 5000 Hz. The study analyzed data from 5 November to 8 November 2018, comprising 50 sets of signal data from five vehicles passing through the monitoring area. The experiments involved detecting vehicle passing and background noise. To ensure the authenticity and reliability of the data, video footage was used to capture the traffic conditions.

## 5. Analysis of Results

### 5.1. Vehicle Detection

**Figure 8:**
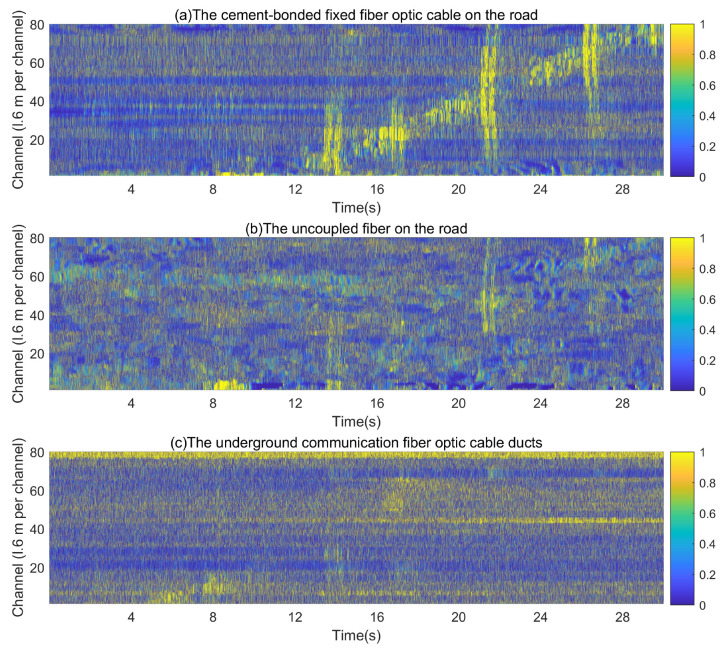
Detection results of a vehicle passing through the detection area (original signal).

Figure 8 presents the results of the distributed acoustic sensing system’s detection when a vehicle passed through the detection area. Vibration signals were recorded on 6 November 2018, at 23:00 during a period of low traffic to minimize the influence of pedestrian activity and other noise on the collected data.

**Figure 9:**
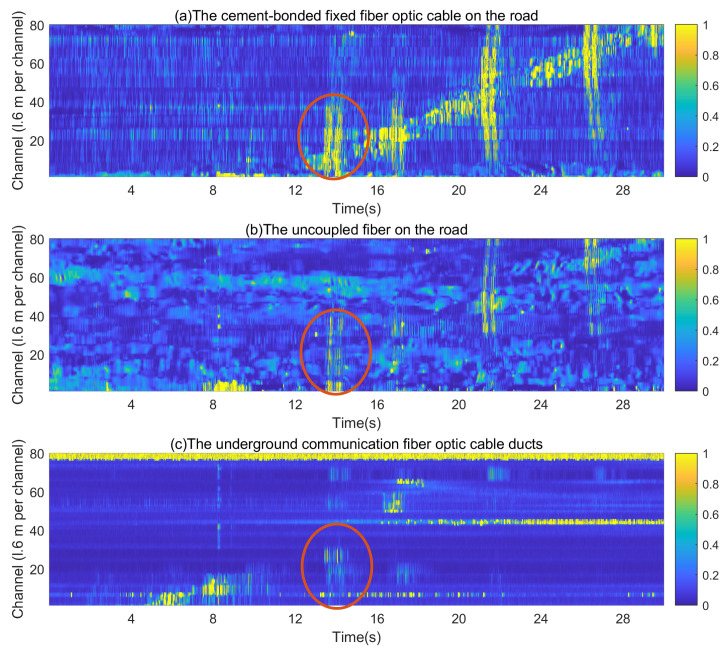
Detection results of a vehicle passing through the detection area (denoised signal).

Figure 9 illustrates the processed results of the proposed improved wavelet threshold denoising algorithm. While a vehicle passes over a speed bump, there is a period of energy transfer through the fiber as illustrated in Figure 9 by the red circle. This period corresponds to the signal generated at the time of vehicle passage.

**Figure 10:**
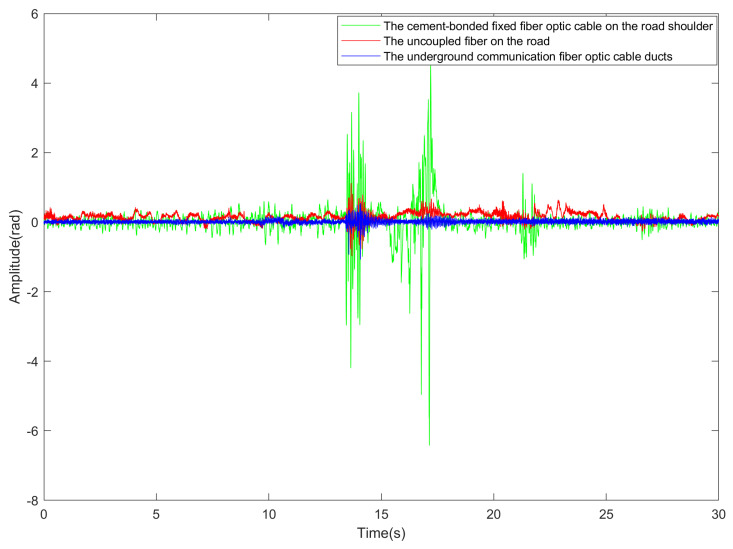
Comparison of vibration data of three different deployment methods (single channel).

**Figure 11:**
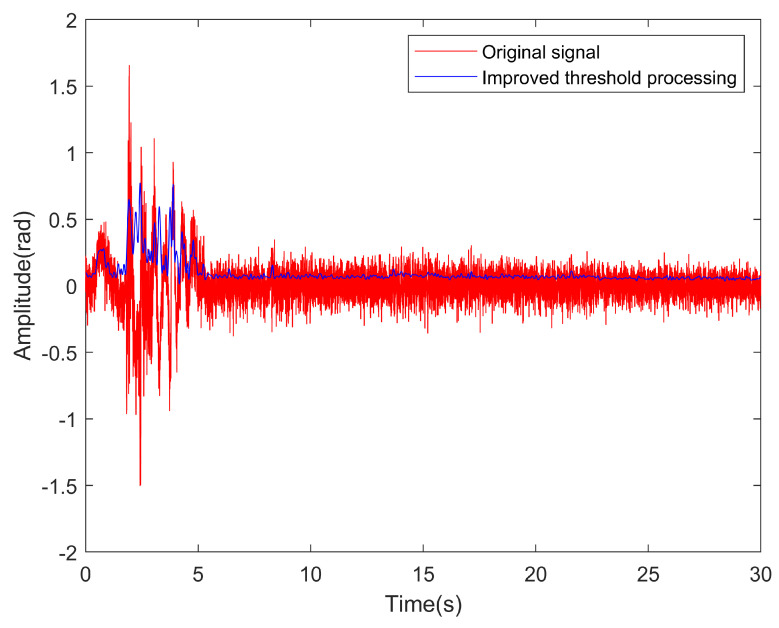
Comparison of vibration data through the detection area before and after denoising (single channel).

Figure 10 compares the vibration data of a single channel collected by three different mounting methods when the vehicle passes through the detection area. Figure 11 illustrates the results of comparing vibration data (single channel) before and after denoising. Notably, the valid vehicle vibration signal corresponds to the continuous signal with a larger amplitude, while the signal with a smaller amplitude represents the irregular noise component.

Based on the video data, the vibration signal represents a vehicle traveling from south to north at a speed of 30 km/h.

During the passage, we calculated the energy and signal-to-noise ratio of selected vehicle channels. To denoise the signal data and determine the energy and signal-to-noise ratio, we employed the improved wavelet thresholding algorithm discussed in Section 3.

#### 5.1.1. Signal-to-Noise Ratio

The signal-to-noise ratio (SNR) is an essential parameter for assessing the quality of seismic data and evaluating the efficacy of denoising techniques. In seismic exploration, the SNR of seismic records is often used as a metric for evaluating data quality. Revised paragraph: A higher Signal-to-Noise Ratio (SNR) value indicates better quality of data and more reliable processing outcomes. In this study, SNR is selected as one of the evaluation metrics to compare the signal quality of fiber-optic vibration signals using three different deployment methods. Revised paragraph: A conventional approach in the time domain is utilized to calculate the SNR, and the root mean square (RMS) is employed as the evaluation metric to improve the precision of the comparison outcomes. The RMS value of the SNR is calculated for various fiber deployments.

The SNR values are computed by dividing the root-mean-square amplitude of a 20 ms window around the maximum amplitude point by the mean RMS amplitude of a 20 ms window of noise at the beginning of the recording. This interval is chosen due to its provision of a background noise level representative of the ambient noise without any vehicular activity. It is noteworthy that the choice of time window could impact the resulting SNR values.

(6) $$SNR_{2}=\frac{RMS_{1}}{RMS_{2}}$$

where $SNR_{2}$ denotes the conventional approach of calculating the signal-to-noise ratio in the time domain; $RMS_{1}$ denotes the root-mean-square (RMS) amplitude of a 20 ms window around the point of maximum amplitude; $RMS_{2}$ denotes the mean RMS amplitude of a 20 ms window of noise at the beginning of the record.

(7) $$SNR_{j}=\sqrt{\frac{\sum_{i=1}^{M}{\left(SNR_{i,j}\right)}^{2}}{M}}$$

(8) $$RMS=\sqrt{\frac{\sum_{j=1}^{N}{\left(SNR_{j}\right)}^{2}}{N}}$$

where i denotes the number of channels of fiber; j denotes the signal data of Group j; $SNR_{i,j}$ denotes the signal-to-noise ratio of channel i of fiber in the signal data of Group j; $SNR_{1}$ denotes the root mean square of the signal-to-noise ratio of the signal data of Group j; and RMS denotes the root mean square of the signal-to-noise ratio of fiber with different deployment methods.

Table 2 displays the signal-to-noise ratio of various fiber deployments. The cement-bonded fixed fiber optic cable on the road shoulder has a signal-to-noise ratio of 71.0034 dB when a vehicle passes through the detection area. Similarly, uncoupled fiber on the road fiber has a signal-to-noise ratio of 14.3719 dB, and the underground communication fiber optic cable ducts exhibit a signal-to-noise ratio of 31.5281 dB. Obviously, the SNR value of the cement-bonded fixed fiber optic cable on the road shoulder is the highest among the three deployment methods. The underground communication fiber optic cable ducts have the second-highest SNR value, and the uncoupled fiber on the road exhibits the lowest SNR value.

#### 5.1.2. Energy

Short Time Energy analysis (STE) is an extensively used and effective technique for energy analysis in the time domain of non-smooth signals in fiber optic communications. The energy of the vehicle vibration signal in the time domain is significantly greater than that of the noise alone.

Revised paragraph: The energy of the vehicle vibration signal in a single channel was computed using a time window of 20 ms, centered on the point of maximum amplitude. This interval was preferred because it provides an accurate representation of the vehicle signal. Different time windows may result in varying energy values, which should be acknowledged. The energy of the fiber using three different deployment methods is expressed in terms of the mean squared difference of the energy in a single channel.

Table 3 showcases the energy levels of various fiber deployments. Upon vehicle passing through the detection area, the cement-bonded fixed fiber optic cable on the road shoulder has an energy of 947.37, while the energy of the uncoupled fiber on the road is 139.50, and the underground communication fiber optic cable ducts have an energy of 94.92. Evidently, the cement-bonded fixed fiber optic cable on the road shoulder has the highest energy while the energy of the uncoupled fiber on the road is second-best and the underground communication fiber optic cable ducts have the lowest energy.

### 5.2. Noise Analysis

**Figure 12:**
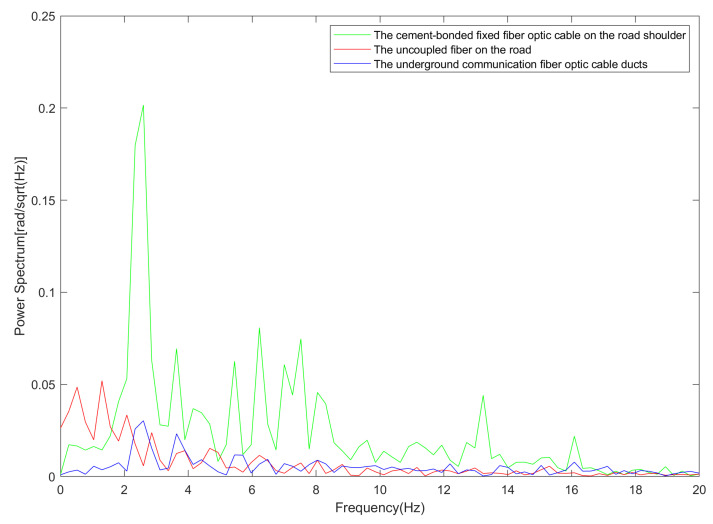
Fiber optic noise spectrum.

The noise spectrum of the cement-bonded fixed fiber optic cable on the road shoulder, the uncoupled fiber on the road, and the underground communication fiber optic cable ducts are displayed in Figure 12. Upon comparison, the noise spectrum peaks of all three deployment methods are primarily concentrated from 0 to 5 Hz; the amplitudes of the three types of fiber optic noise are notably greater at lower frequencies. The primary frequency of the uncoupled fiber on the road is close to zero, displaying a continuous decay, which implies that lower frequency coupling is more effective. The cement-bonded fixed fiber optic cable on the road shoulder has a single peak and a more pronounced jump. The underground communication fiber optic cable ducts have dual peaks and more evident attenuation.

The primary frequencies of the cement-bonded fixed fiber optic cable on the road shoulder and underground communication fiber optic cable ducts are nearer to the primary frequency of the vehicle passing via the detection area. This infers that the coupling quality of the cement-bonded fixed fiber optic cable on the road shoulder and underground communication fiber optic cable ducts surpasses that of the uncoupled fiber on the road fiber within the primary vehicle frequency. The amplitude of the cement-bonded fixed fiber optic cable on the road shoulder is significantly higher than that of the underground communication fiber optic cable ducts. This indicates that the coupling quality of the cement-bonded fixed fiber optic cable on the road shoulder exceeds that of the underground communication fiber optic cable ducts.

## 6. Conclusions

This paper presents the usage of an improved wavelet threshold algorithm to compare the signal quality of three different fiber optic placement methods. The effectiveness of the algorithm is validated through comparisons of signal-to-noise ratio (SNR) and root-mean-square (RMS) errors produced by different threshold denoising algorithms. The experiments were conducted using a DAS (distributed acoustic sensing) system on the campus of Beijing Jiaotong University, where vibration signals induced by vehicle motion were detected through the uncoupled fiber on the road, the underground communication fiber optic cable ducts, and the cement-bonded fixed fiber optic cable on the road shoulder.

The experiment results from the three different placement methods were then analyzed using the improved wavelet threshold algorithm. The comparison of SNR, energy, and noise analysis were conducted to compare the difference in vehicle signals picked up by each fiber optic placement method. The experimental results demonstrate that the improved wavelet threshold denoising algorithm yields superior denoising outcomes compared to the conventional wavelet algorithm. The signal-to-noise ratio (SNR) and energy levels obtained from the cement-bonded fixed fiber optic cable on the road shoulder are the highest. The obtained vehicle vibration signal’s quality from the cement-bonded fixed fiber optic cable on the road shoulder is the best. The ground layer filters a portion of the signal, leading to a lower amount of received vehicle signal and noise by the underground communication fiber optic cable ducts. Therefore, the signal-to-noise ratio (SNR) of the underground communication fiber optic cable ducts is higher than that of the uncoupled fiber on the road. However, the energy of the signal in the uncoupled fiber on the road is higher than that in the underground communication fiber optic cable ducts. As illustrated in Figure 8, the underground communication fiber may result in a signal loss in certain channels due to weak coupling, causing the signals to be undetectable.

Therefore, the cement-bonded fixed fiber optic cable on the road shoulder is preferred for practical applications, followed by the uncoupled fiber on the road, with the underground communication fiber optic cable ducts as the last resort.

In future research, we will compare additional diverse fiber optic deployment methods and develop complete theoretical models to determine the optimal approach for using distributed acoustic sensing across different industries, with the goal of maximizing efficiency. However, this study identified the following limitations: (1) The study was limited to a university campus, and the environment, including the road surface material, may have affected the fiber optic coupling. (2) The fiber optic deployment distance was short, and the sample size was small. (3) The experimental conditions were limited to straight-line travel at a constant speed.

## Figures and Tables

**Table 1:** Signal-to-noise ratio and root-mean-square error of different threshold denoising algorithms.

Denoising Algorithm	Hard Threshold	Soft Threshold	Improved Thresholds
Signal-to-noise ratio (SNR)	5.4782	21.3241	23.5637
Root mean square error (RMSE)	1.0231	0.5298	0.4054

**Table 2:** Signal-to-noise ratio of different deployments of fiber.

Deployment Method	Cement-Bonded Fixed Fiber Optic Cable on the Road Shoulder	Uncoupled Fiber on the Road	Underground Communication Fiber Optic Cable Ducts
RMS	71.0034	14.3719	31.5281

**Table 3:** Energy of different deployments for different deployments of optical fiber.

Deployment Method	Cement-Bonded Fixed Fiber Optic Cable on the Road Shoulder	Uncoupled Fiber on the Road	Underground Communication Fiber Optic Cable Ducts
ENERGY	947.37	139.50	94.92

## Data Availability

The data presented in this study are available on request from the corresponding author on reasonable request.

## References

[1] Dimitrakopoulos G., Demestichas P. (2010). Intelligent Transportation Systems. IEEE Veh. Technol. Mag..

[2] Qu F.Z., Wang F.Y., Yang L.Q. (2010). Intelligent Transportation Spaces: Vehicles, Traffic, Communications, and Beyond. IEEE Commun. Mag..

[3] Park S.J., Kim T.Y., Kang S.M., Koo K.H. (2003). A novel signal processing technique for vehicle detection radar. IEEE MTT-S Int. Microw. Symp. Dig..

[4] Jeng S.L., Chieng W.H., Lu H.P. (2014). Estimating Speed Using a Side-Looking Single-Radar Vehicle Detector. IEEE Trans. Intell. Transp. Syst..

[5] Der S., Chan A., Nasrabadi N., Kwon H. (2004). Automated vehicle detection in forward-looking infrared imagery. Appl. Opt..

[6] Arshad N.M., Misnan M.F., Razak N.A. Single infra-red sensor technique for line-tracking autonomous mobile vehicle. Proceedings of the 2011 IEEE 7th International Colloquium on Signal Processing and its Applications.

[7] Zhang C., Dong H.H., Jia L.M., Qin Y., Yang Z.Y. Robust Vehicle Detection and Identification with Single Magnetic Sensor. Proceedings of the 2017 2nd IEEE International Conference on Intelligent Transportation Engineering (ICITE).

[8] Wang Q., Zheng J.Y., Xu H., Xu B., Chen R. (2018). Roadside Magnetic Sensor System for Vehicle Detection in Urban Environments. IEEE Trans. Intell. Transp. Syst..

[9] Zhou J., Gao D.S., Zhang D. (2007). Moving vehicle detection for automatic traffic monitoring. IEEE Trans. Veh. Technol..

[10] Jazayeri A., Cai H.Y., Zheng J.Y., Tuceryan M. (2011). Vehicle Detection and Tracking in Car Video Based on Motion Model. IEEE Trans. Intell. Transp. Syst..

[11] Mishra D., Asutkar G. (2013). Vehicle detection and classification using wireless sensor network. Int. J. Adv. Res. Electr. Electron. Instrum. Eng..

[12] Aljaafreh A., Al Assaf A. (2013). A Wireless Sensor Network Design and Implementation for Vehicle Detection, Classification, and Tracking. Airborne Intelligence, Surveillance, Reconnaissance (ISR) Systems and Applications X.

[13] Parker T., Shatalin S., Farhadiroushan M. (2014). Distributed Acoustic Sensing—A new tool for seismic applications. First Break.

[14] Molenaar M.M., Hill D.J., Webster P., Fidan E., Birch B. (2012). First Downhole Application of Distributed Acoustic Sensing for Hydraulic-Fracturing Monitoring and Diagnostics. SPE Hydraul. Fract. Technol. Conf. Exhib..

[15] Owen A., Duckworth G., Worsley J. OptaSense: Fibre optic distributed acoustic sensing for border monitoring. Proceedings of the 2012 European Intelligence and Security Informatics Conference.

[16] Wang B., Zeng X., Song Z., Li X., Yang J. (2021). Seismic observation and subsurface imaging using an urban telecommunication optic-fiber cable. Chin. Sci. Bull..

[17] Song Z., Zeng X., Wang B., Yang J., Li X., Wang H.F. (2021). Distributed acoustic sensing using a large-volume airgun source and internet fiber in an urban area. Seismol. Res. Lett..

[18] Zeng X.F., Bao F., Thurber C.H., Lin R.B., Wang S.F., Song Z.H., Han L.B. (2022). Turning a Telecom Fiber-Optic Cable into an Ultradense Seismic Array for Rapid Post-earthquake Response in an Urban Area. Seismol. Res. Lett..

[19] Martin E., Lindsey N., Dou S., Ajo-Franklin J., Daley T., Freifeld B., Robertson M., Ulrich C., Wagner A., Bjella K. (2016). Interferometry of a roadside DAS array in Fairbanks, AK. 2016 SEG International Exposition and Annual Meeting.

[20] Cai Y.P., Ma J.H., Yan W.F., Zhang W.Y., An Y.H. (2021). Aircraft Detection Using Phase-Sensitive Optical-Fiber OTDR. Sensors.

[21] Liu H.Y., Ma J.H., Yan W.F., Liu W.S., Zhang X., Li C.C. (2018). Traffic Flow Detection Using Distributed Fiber Optic Acoustic Sensing. IEEE Access.

[22] Liu H.Y., Ma J.H., Xu T.W., Yan W.F., Ma L.L., Zhang X. (2020). Vehicle Detection and Classification Using Distributed Fiber Optic Acoustic Sensing. IEEE Trans. Veh. Technol..

[23] Cai Y.P., Yan W.F., Liu H.Y., Sun Y.T., Zhou X.L. (2020). Security Monitoring of Smart Campus Using Distributed Fiber Optic Acoustic Sensing. AOPC 2020: Optical Information and Network.

[24] Xie T., Zhang C.-C., Chen J.-S., Fu Y.-P., Dai L., Yin J., Shi B. (2020). Influence of fiber-optic cable structure, sand density, and acoustic frequency on DAS amplitude response: Preliminary results. IOP Conference Series: Earth and Environmental Science.

[25] Chen J.Y., Ning J.R., Chen W.C., Wang X.K., Wang W., Zhang G.L. (2019). Distributed acoustic sensing coupling noise removal based on sparse optimization. Interpretation.

[26] Munn J.D., Coleman T.I., Parker B.L., Mondanos M.J., Chalari A. (2017). Novel cable coupling technique for improved shallow distributed acoustic sensor VSPs. J. Appl. Geophys..

[27] Fiber Optic Sensing Association (2018). Installation Considerations for Highways. https://www.academia.edu/36932622/Installation_Considerations_of_Highway_Guardrails.

[28] Masoudi A., Belal M., Newson T.P. (2013). A distributed optical fibre dynamic strain sensor based on phase-OTDR. Meas. Sci. Technol..

[29] Bao X.Y., Chen L. (2012). Recent Progress in Distributed Fiber Optic Sensors. Sensors.

[30] Sun Q.Z., Ai F., Liu D.M., Cheng J.W., Luo H.B., Peng K., Luo Y.Y., Yan Z.J., Shum P.P. (2017). M-OTDR sensing system based on 3D encoded microstructures. Sci. Rep..

[31] Taylor H.F., Lee C.E. (1993). Apparatus and Method for Fiber Optic Intrusion Sensing. U.S. Patent.

[32] Yang G.Y., Fan X.Y., Liu Q.W., He Z.Y. (2018). Frequency Response Enhancement of Direct-Detection Phase-Sensitive OTDR by Using Frequency Division Multiplexing. J. Lightwave Technol..

[33] Lu Y.L., Zhu T., Chen L.A., Bao X.Y. (2010). Distributed Vibration Sensor Based on Coherent Detection of Phase-OTDR. J. Lightwave Technol..

[34] Dandridge A., Tveten A.B., Giallorenzi T.G. (1982). Homodyne demodulation scheme for fiber optic sensors using phase generated carrier. IEEE Trans. Microw. Theory Tech..

